# A difference-based approach in the partially linear model with dependent errors

**DOI:** 10.1186/s13660-018-1857-x

**Published:** 2018-10-01

**Authors:** Zhen Zeng, Xiangdong Liu

**Affiliations:** Department of Statistics, Jinan University, Guangzhou, P.R. China

**Keywords:** 62G05, 62G20, NSD random variables, Partially linear model, Asymptotic normality, Finite difference, Least square

## Abstract

We study asymptotic properties of estimators of parameter and non-parameter in a partially linear model in which errors are dependent. Using a difference-based and ordinary least square (DOLS) method, the estimator of an unknown parametric component is given and the asymptotic normality of the DOLS estimator is obtained. Meanwhile, the estimator of a nonparametric component is derived by the wavelet method, and asymptotic normality and the weak convergence rate of the wavelet estimator are discussed. Finally, the performance of the proposed estimator is evaluated by a simulation study.

## Introduction

Consider the partially linear model (PLM)

1 $$\begin{aligned} y_{i}=x_{i}^{T}\beta+f(t_{i})+e_{i},\quad 1 \leq i\leq n, \end{aligned}$$

where the superscript *T* denotes the transpose, $y_{i}$ are scalar response variables, $x_{i}=(x_{i1},\ldots,x_{id})^{T}$ are explanatory variables, *β* is a *d*-dimensional column vector of the unknown parameter, $f(\cdot)$ is an unknown function, $t_{i}$ are deterministic with $0\leq t_{1}\leq\cdots\leq t_{n}\leq1$, and $e_{i}$ are random errors.

PLM was first considered by Engle et al. [1], and now is one of the most widely used statistical models. It can be applied in almost every field, such as engineering, economics, medical sciences and ecology, etc. There are many authors (see [2–8]) concerned with various estimation methods to obtain estimators of the unknown parameters and nonparameters for partially linear model. Deep results such as asymptotic normality of estimators have been obtained.

In this paper, by a difference-based approach, we will use the ordinary least square and wavelet to investigate model (1). The differencing procedures provide a convenient means for introducing nonparametric techniques to practitioners in a way which parallels their knowledge of parametric techniques, and differencing procedures may easily be combined with other procedures. For example, Wang et al. [9] obtained a difference-based approach to the semiparametric partially linear model. Tabakan et al. [10] studied a difference-based ridge in partially linear model. Duran et al. [11] investigated the difference-based ridge and Liu type estimators in semiparametric regression models. Hu et al. [12] used a difference-based Huber Dutter estimator (DHD) to obtain the root variance *σ* and parametric *β* for partially linear model. Wu [13] constructed the restricted difference-based Liu estimator for the parametric component of partially linear model. However, in the majority of the previous work it is assumed that errors are independent. The asymptotic problem of difference-based estimators of partially linear model with dependent errors is in practice important. In this paper, we use a difference-based and ordinary least square method to study the partially linear model with dependent errors.

For the dependent errors $e_{i}$ we confine ourselves to negatively superadditive dependent (NSD) random variables. There are many applications of NSD random variables in multivariate statistical analysis; see [14–23]. Hence, it is meaningful to study the properties of NSD random variables. The formal definition of NSD random variables is the following.

### Definition 1

(Kemperman [24])

A function Φ: $\mathbf{R}^{n}\rightarrow\mathbf{R}$ is called superadditive if $\Phi (x\vee y ) + \Phi (x\wedge y )\geq\Phi(x) + \Phi (y )$ for all $x, y \in\mathbf{R}^{n}$, where ∨ stands for componentwise maximum, and ∧ for componentwise minimum.

### Definition 2

(Hu [25])

A sequence $\{e_{1}, e_{2},\ldots, e_{n}\}$ is said to be NSD if

2 $$\begin{aligned} E\Phi (e_{1}, e_{2},\ldots, e_{n} )\leq E \Phi(Y_{1}, Y_{2},\ldots, Y_{n}), \end{aligned}$$

where $Y_{1}, Y_{2},\ldots, Y_{n}$ are independent with $e_{i}\stackrel{d}{=}Y_{i}$ for each *i*, and Φ is a superadditive function such that the expectations in (2) exist. An infinite sequence $\{e_{n}, n\geq1\}$ of random variables is said to be NSD if $\{e_{1}, e_{2},\ldots, e_{n}\}$ is NSD for all $n\geq1$.

In addition, using the wavelet method (see [26–29]), the weak convergence rate and asymptotic normality of the estimator of $f(\cdot)$ are obtained.

Throughout the paper we fix the following notations. $\beta_{0}$ is the true value of the unknown parameter *β*. **Z** is the set of integers, **N** is the set of natural numbers, **R** is the set of real numbers. Denote $x^{+}=\max(x,0)$, and $x^{-}=(-x)^{+}$. Let $C_{1},C_{2}, C_{3}, C_{4}$ are positive constants. For a sequence of random variables $\eta_{n}$ and a positive sequence $d_{n}$, write $\eta_{n}=o(d_{n})$ if $\eta_{n}/d_{n}$ converges to 0 and $\eta_{n}=O (d_{n})$ if $\eta_{n}/d_{n}$ is bounded. We can similarly define the notations of $o_{P}$ and $O_{P}$ for stochastic convergence and stochastic bounded. Weak convergence of a distribution is denoted by $H_{n}\stackrel{D}{\rightarrow}H$, and for random variables by $Y_{n}\stackrel{D}{\rightarrow}Y$. $\|x\|$ is the Euclidean norm of *x*, and $\lfloor x\rfloor=\max\{k\in\mathbf {Z}:k\leq x\}$.

## Estimation method

Define the $(n-m)\times n$ differencing matrix *D* as

$$D=\left(\begin{array}{cccccccccccc} d_{0} & d_{1} & d_{2} & \cdots & d_{m} & 0 & \cdots & \cdots & \cdots & \cdots & \cdots & 0\\ 0 & d_{0} & d_{1} & d_{2} & \cdots & d_{m} & 0 & \cdots & \cdots & \cdots & \cdots & 0\\ \vdots & \vdots & \vdots & \vdots & \vdots & \vdots & \vdots & \vdots & \vdots & \vdots & \vdots & \vdots \\ 0 & \cdots & \cdots & \cdots & \cdots & 0 & d_{0} & d_{1} & d_{2} & \cdots & \cdots & 0\\ 0 & \cdots & \cdots & \cdots & \cdots & \cdots & 0 & d_{0} & d_{1} & d_{2} & \cdots & d_{m} \end{array}\right),$$

where the positive integer number *m* is the order of differencing and $d_{0},d_{1},\ldots,d_{m} $ are differencing weights satisfying

3 $$\begin{aligned} \sum_{q=0}^{m} d_{q}=0,\qquad \sum _{q=0}^{m} d^{2}_{q}=1. \end{aligned}$$

This differencing matrix is given by Yatchew [30]. Using the differencing matrix to model (1), we have

4 $$\begin{aligned} DY=DX\beta+Df+De. \end{aligned}$$

From Yatchew [30], the application of differencing matrix *D* in model (1) can remove the nonparametric effect in large samples, so we will ignore the presence of *Df*. Thus, we can rewrite (4) as

5 $$\begin{aligned} \tilde{Y}=\tilde{X}\beta+\tilde{e}, \end{aligned}$$

where $\tilde{Y}= (\tilde{y}_{1},\ldots,\tilde{y}_{n-m} )^{T}$, $\tilde{X}= (\tilde{x}_{1},\ldots,\tilde{x}_{n-m} )^{T}$ and $\Sigma_{n}=\tilde{X}^{T}\tilde{X}$ is nonsingular for large *n*, $\tilde{e}= (\tilde{e}_{1},\ldots,\tilde{e}_{n-m} )^{T}$, $\tilde{y}_{i}=\sum_{q=0}^{m}d_{q}y_{i+q}$, $\tilde{x}_{i}=\sum_{q=0}^{m}d_{q}x_{i+q}$, $\tilde{e}_{i}=\sum_{q=0}^{m}d_{q}e_{i+q}$, $i=1,\ldots,n-m$.

As a usual regression model, the ordinary least square estimator $\hat{\beta}_{n}$ of the unknown parameter *β* is given as

6 $$\begin{aligned} \hat{\beta}_{n}=\arg\min_{\beta}\sum _{i=1}^{n-m}\bigl(\tilde{y}_{i}-\tilde {x}^{T}_{i}\beta\bigr)^{2}. \end{aligned}$$

Then the estimator satisfies

$$\begin{aligned} -2\sum_{i=1}^{n-m}\tilde{x}_{i} \bigl(\tilde{y}_{i}-\tilde{x}^{T}_{i}\hat{ \beta}_{n}\bigr)=0, \end{aligned}$$

and hence

7 $$\begin{aligned} \hat{\beta}_{n}=\Sigma_{n}^{-1} \tilde{X}^{T}\tilde{Y}. \end{aligned}$$

In the following, we use wavelet techniques to estimate $f(\cdot)$ if $\hat{\beta}_{n}$ is known.

Suppose that there exists a scaling function $\phi(\cdot)$ in the Schwartz space $S_{l}$ and a multiresolution analysis $\{V_{\tilde{m}}\}$ in the concomitant Hilbert space $L^{2}(\mathbf{R})$, with the reproducing kernel $E_{\tilde{m}}(t,s)$ given by

$$\begin{aligned} E_{\tilde{m}}(t,s)=2^{\tilde{m}}E_{0}\bigl(2^{\tilde{m}}t, 2^{\tilde {m}}s\bigr)=2^{\tilde{m}}\sum_{k\in\mathbf{Z}} \phi\bigl(2^{\tilde{m}}t-k\bigr)\phi \bigl(2^{\tilde{m}}s-k\bigr). \end{aligned}$$

Let $A_{i}=[s_{i-1}, s_{i}]$ denote intervals that partition $[0, 1]$ with $t_{i} \in A_{i}$ for $1\leq i\leq n$. Then the estimator of the nonparameter $f(t)$ is given by

8 $$\begin{aligned} \hat{f}_{n}(t)=\sum_{i=1}^{n} \bigl(y_{i}-x^{T}_{i}\hat{\beta}_{n} \bigr) \int_{A_{i}}{E_{\tilde {m}}(t,s)}\,ds. \end{aligned}$$

## Preliminary conditions and lemmas

In this section, we give the following conditions and lemmas which will be used to obtain the main results.

1. $\max_{1\leq i \leq n}\|x_{i}\|=C_{1}<\infty$.
2. $f(\cdot)\in H^{\alpha}$ (Sobolev space), for some $\alpha>1/2$.
3. $f(\cdot)$ is Lipschitz function of order $\gamma>0$.
4. $\phi(\cdot)$ belongs to $S_{l}$, which is a Schwartz space for $l\geq\alpha$. $\phi(\cdot)$ is a Lipschitz function of order 1 and has compact support, in addition to $|\hat{\phi }(\xi)-1|=O(\xi)$ as $\xi\rightarrow0$, where *ϕ̂* denotes Fourier transform of *ϕ*.
5. $s_{i}$, $1\leq i\leq n$, satisfy $\max_{1\leq i\leq n}(s_{i}-s_{i-1})=O(n^{-1})$, and $2^{\tilde{m}}=O(n^{1/3})$.

### Remark 3.1

Condition (C1) is standard and often imposed in the estimator of partial linear models, once can refer to Zhao et al. [31]. Conditions (C2)–(C5) are used by Hu et al. [29]. Therefore, our conditions are very mild and can easily be satisfied.

### Lemma 3.1

(Hu [25])

*Suppose that* $\{e_{1}, e_{2}, \ldots, e_{n}\}$ *is NSD*.

(i) *If* $g_{1},g_{2},\ldots,g_{n}$ *are nondecreasing functions*, *then* $\{ g_{1}(e_{1}),g_{2}(e_{2}),\ldots,g_{n}(e_{n})\}$ *is NSD*.
(ii) *For any* $2\leq m\leq n$ *and* $1\leq i_{1}< i_{2}<\cdots<i_{m}$, $\{ e_{i_{1}}, e_{i_{2}}, \ldots, e_{i_{m}}\}$ *is NSD*.

### Lemma 3.2

(Wang et al. [17])

*Let* $p>1$. *Let* $\{e_{n},n \geq1\}$ *be a sequence of NSD random variables with* $Ee_{n}=0$ *and* $E|e_{n}|^{p}<\infty$ *for each* $n \geq1$. *Then for all* $n \geq1$,

9 $$\begin{aligned} E \Biggl(\max_{1\leq k\leq n} \Biggl\vert \sum _{i=1}^{k}e_{i} \Biggr\vert ^{p} \Biggr)\leq 2^{3-p}\sum_{i=1}^{n}E \vert e_{i} \vert ^{p} \quad\textit{for } 1< p\leq2 \end{aligned}$$

*and*

10 $$\begin{aligned} E \Biggl(\max_{1\leq k\leq n} \Biggl\vert \sum _{i=1}^{k}e_{i} \Biggr\vert ^{p} \Biggr)\leq 2 \biggl(\frac{15p}{\ln p} \biggr)^{p} \Biggl[\sum_{i=1}^{n} E \vert e_{i} \vert ^{p}+ \Biggl(\sum _{i=1}^{n}Ee_{i}^{2} \Biggr) ^{p/2} \Biggr]\quad \textit{for } p> 2. \end{aligned}$$

### Lemma 3.3

*Let* $p>1$. *Let* $\{e_{n},n \geq1\}$ *be a sequence of NSD random variables with* $Ee_{n}=0$ *and* $E|e_{n}|^{p}<\infty$ *for all* $n\geq1$, *and* $\{c_{q}, 0\leq q\leq m\}$ *be a sequence of real constants*. *Then for all* $n\geq1$,

11 $$\begin{aligned} E \Biggl(\max_{1\leq k\leq n-m} \Biggl\vert \sum _{i=1}^{k}\sum_{q=0}^{m}c_{q}e_{i+q} \Biggr\vert ^{p} \Biggr)\leq4m^{p-1}\sum _{i=1}^{n}\sum_{q=0}^{m}E \vert c_{q}e_{i+q} \vert ^{p} \quad\textit{for } 1< p \leq2 \end{aligned}$$

*and*, *for* $p>2$,

12 $$\begin{aligned} & E \Biggl(\max_{1\leq k\leq{n-m}} \Biggl\vert \sum _{i=1}^{k}\sum_{q=0}^{m}c_{q}e_{i+q} \Biggr\vert ^{p} \Biggr) \\ &\quad\leq 2^{p+1}m^{p-1} \biggl(\frac{15p}{\ln p} \biggr)^{p} \Biggl[\sum_{i=1}^{n} \sum_{q=0}^{m}E \vert c_{q}e_{i+q} \vert ^{p}+ \Biggl(\sum_{i=1}^{n} \sum_{q=0}^{m}E(c_{q}e_{i+q})^{2} \Biggr)^{p/2} \Biggr]. \end{aligned}$$

### Proof

Let $z_{1i}=\sum^{m}_{q=0}c^{+}_{q}e_{i+q}$, $z_{2i}=\sum^{m}_{q=0}c^{-}_{q}e_{i+q}$, then $\sum_{q=0}^{m}c_{q} e_{i+q}=z_{1i}-z_{2i}$, and $\{c^{+}_{q}e_{i+q}, i\geq1\}$ and $\{c^{-}_{q}e_{i+q},i\geq1\}$ are both NSD random variables for all $0\leq q\leq m$ by Lemma 3.1. By the $C_{r}$-inequality,

$$\begin{aligned} & E \Biggl(\max_{1\leq k\leq n-m} \Biggl\vert \sum _{i=1}^{k}\sum_{q=0}^{m}c_{q} e_{i+q} \Biggr\vert ^{p} \Biggr) \\ &\quad= E \Biggl(\max_{1\leq k\leq n-m} \Biggl\vert \sum _{i=1}^{k}(z_{1i}-z_{2i}) \Biggr\vert ^{p} \Biggr) \\ &\quad\leq 2^{p-1} \Biggl\{ E \Biggl(\max_{1\leq k\leq n-m} \Biggl\vert \sum_{i=1}^{k}z_{1i} \Biggr\vert ^{p} \Biggr)+E \Biggl(\max_{1\leq k\leq n-m} \Biggl\vert \sum_{i=1}^{k}z_{2i} \Biggr\vert ^{p} \Biggr) \Biggr\} \\ &\quad\leq 2^{p-1}m^{p-1}\sum_{q=0}^{m} \Biggl\{ E \Biggl(\max_{1\leq k\leq n-m} \Biggl\vert \sum _{i=1}^{k}c^{+}_{q}e_{i+q} \Biggr\vert ^{p} \Biggr)+E \Biggl(\max_{1\leq k\leq n-m} \Biggl\vert \sum_{i=1}^{k}c^{-}_{q}e_{i+q} \Biggr\vert ^{p} \Biggr) \Biggr\} . \end{aligned}$$

In the case $1< p\leq2$, it follows from Lemma 3.2 that

13 $$\begin{aligned} & E \Biggl(\max_{1\leq k\leq n-m} \Biggl\vert \sum _{k=1}^{k}\sum_{q=0}^{m}c_{q} e_{i+q} \Biggr\vert ^{p} \Biggr) \\ &\quad\leq 2^{p-1}m^{p-1}\sum_{q=0}^{m} \Biggl\{ E \Biggl(\max_{1\leq k\leq n-m} \Biggl\vert \sum _{i=1}^{k}c^{+}_{q}e_{i+q} \Biggr\vert ^{p} \Biggr)+E \Biggl(\max_{1\leq k\leq n-m} \Biggl\vert \sum_{i=1}^{k}c^{-}_{q}e_{i+q} \Biggr\vert ^{p} \Biggr) \Biggr\} \\ &\quad \leq 4m^{p-1} \Biggl(\sum_{i=1}^{n-m} \sum_{q=0}^{m}E \bigl\vert c^{+}_{q}e_{i+q} \bigr\vert ^{p}+\sum _{i=1}^{n-m}\sum_{q=0}^{m}E \bigl\vert c^{-}_{q}e_{i+q} \bigr\vert ^{p} \Biggr). \end{aligned}$$

Note that $|c_{q}|^{p}=|c_{q}^{+}|^{p}+|c_{q}^{-}|^{p}$, the desired result (11) follows from (13) immediately. In the same way, we also have (12). The proof is completed. □

### Remark 3.2

From Lemma 3.3 and Lemma 3.1, we have, for $1< p\leq2$,

14 $$\begin{aligned} E \Biggl( \Biggl\vert \sum_{i\in S}\sum _{q=0}^{m}c_{q}e_{i+q} \Biggr\vert ^{p} \Biggr)\leq 4m^{p-1} \Biggl\{ \sum _{i\in S}\sum_{q=0}^{m}E \vert c_{q}e_{i+q} \vert ^{p} \Biggr\} \end{aligned}$$

and, for $p>2$,

15 $$\begin{aligned} & E \Biggl( \Biggl\vert \sum_{i\in S}\sum _{q=0}^{m}c_{q}e_{i+q} \Biggr\vert ^{p} \Biggr) \\ &\quad \leq 2^{p+1}m^{p-1} \biggl(\frac{15p}{\ln p} \biggr)^{p} \Biggl[\sum_{i\in S} \sum _{q=0}^{m}E \vert c_{q}e_{i+q} \vert ^{p}+ \Biggl(\sum_{i\in S}\sum _{q=0}^{m}E(c_{q}e_{i+q})^{2} \Biggr)^{p/2} \Biggr], \end{aligned}$$

where $S\subset\{1,2,\ldots,n\}$.

### Lemma 3.4

*Let* *A* *and* *B* *be disjoint subsets of* **N**, *and* $\{X_{j}, j\in A\cup B\}$ *be a sequence of NSD random variables*. *Let* *f*: $\mathbf{R}\rightarrow\mathbf{R}$ *and* *g*: $\mathbf {R}\rightarrow\mathbf{R}$ *be differentiable with bounded derivatives*, *and* $\Vert \cdot \Vert _{\infty}$ *stand for supnorm*. *Then*

$$\begin{aligned} & \biggl\vert \operatorname{Cov} \biggl\{ f \biggl(\sum_{i\in A}a_{i}X_{i} \biggr),g \biggl(\sum_{j\in A}a_{j}X_{j} \biggr) \biggr\} \biggr\vert \leq \bigl\Vert f' \bigr\Vert _{\infty}\bigl\Vert g' \bigr\Vert _{\infty}\biggl\vert \operatorname{Cov} \biggl(\sum_{i\in A}a_{i}X_{i}, \sum_{j\in B}a_{j}X_{j} \biggr) \biggr\vert , \end{aligned}$$

*provided the covariation on the right hand side exists*, *where* $\{a_{i}, 1\leq i \leq n\}$ *is an array of real numbers*.

### Proof

For a pair of random variables $Z_{1}=\sum_{i\in A}a_{i}X_{i}$, $Z_{2}=\sum_{j\in B}a_{j}X_{j}$, we have

$$\begin{aligned} H(z_{1},z_{2})=P(Z_{1}\leq z_{1}, Z_{2}\leq z_{2})-P(Z_{1}\leq z_{1})P(Z_{2} \leq z_{2}). \end{aligned}$$

Denote by $F(z_{1},z_{2})$ the joint distribution functions of $(Z_{1},Z_{2})$, and $F_{Z_{1}}({z_{2}}),F_{Z_{2}}({z_{2}})$ the marginal distribution function of ${Z_{1}},{Z_{2}}$, one gets

$$\begin{aligned} \operatorname{Cov}(Z_{1},Z_{2})&=E(Z_{1}Z_{2})-E(Z_{1})E(Z_{2}) \\ & = \int \!\!\int\bigl[F(z_{1},z_{2})-F_{Z_{1}}(z_{1})F_{Z_{2}}(z_{2}) \bigr]\,dz_{1}\,dz_{2}= \int\!\! \int H(z_{1},z_{2})\,dz_{1} \,dz_{2}, \end{aligned}$$

this relation was established in Lehmann [32] for any two random variables $Z_{1}$ and $Z_{2}$ with $\operatorname{Cov}(Z_{1},Z_{2})$ exist. Let $f,g$ are complex valued function on **R** with derivatives $f',g'<\infty$, then we have

$$\begin{aligned} & \bigl\vert \operatorname{Cov}\bigl(f(Z_{1}),g(Z_{2})\bigr) \bigr\vert \\ &\quad = \int\!\! \int f'(Z_{1})g'(Z_{2})H(z_{1},z_{2}) \, dz_{1}\,dz_{2} \\ &\quad \leq \int\!\! \int \bigl\vert f'(Z_{1}) \bigr\vert \bigl\vert g'(Z_{2}) \bigr\vert \bigl\vert H(z_{1},z_{2}) \bigr\vert \,dz_{1} \,dz_{2}\leq \bigl\Vert f' \bigr\Vert _{\infty}\bigl\Vert g' \bigr\Vert _{\infty}\bigl\vert \operatorname{Cov}(Z_{1},Z_{2}) \bigr\vert . \end{aligned}$$

The proof is completed. □

### Lemma 3.5

*Let* $\{e_{n}, n\geq1\}$ *be a sequence of NSD random variable with* $Ee_{n}=0$. *Let* $\tilde{e}_{i_{j}}=\sum_{q=0}^{m}d_{q}e_{i_{j}+q}$, *and* $|i_{j}-i_{k}|>m$ *if* $j\neq k$. *Then*

16 $$\begin{aligned} \Biggl\vert E\exp \Biggl(\mathrm{i}\sum_{j=1}^{n}t_{i_{j}} \tilde {e}_{i_{j}} \Biggr)-\prod_{j=1}^{n}E \exp (\mathrm{i}t_{i_{j}}\tilde {e}_{i_{j}} ) \Biggr\vert \leq- \sum_{ j=1}^{n}\sum _{k=j+1}^{n}\sum_{q1=0}^{m} \sum_{q2=0}^{m}t^{2}_{0}\operatorname{Cov} (e_{i_{j}+q1},e_{i_{k}+q2} ), \end{aligned}$$

*where* $\mathrm{i}=\sqrt{-1}$, $\sum_{q=0}^{m}d_{q}=0$ *and* $\sum_{q=0}^{m}d^{2}_{q}=1$, $t_{i_{1}}, t_{i_{2}}, \ldots, t_{i_{n}}$ *are real numbers with* $|t_{i_{j}}|\leq t_{0}$.

### Proof

Notice that the result is true for $n=1$.

For $n=2$, let $f(\tilde{e}_{i_{1}})=\exp\{\mathrm{i}t_{i_{1}}\tilde {e}_{i_{1}}\}, g(\tilde{e}_{i_{2}})=\exp\{\mathrm{i}t_{i_{2}}\tilde{e}_{i_{2}}\} $. Then, by Lemma 3.4 and $\sum_{q=0}^{m}d_{q}^{2}=1$,

$$\begin{aligned} &\bigl\vert E\exp \{\mathrm{i}t_{i_{1}}\tilde{e}_{i_{1}}+ \mathrm {i}t_{i_{2}}\tilde{e}_{i_{2}} \}-E\exp \{ \mathrm{i}t_{i_{1}}\tilde {e}_{i_{1}} \}E\exp \{ \mathrm{i}t_{i_{2}}\tilde{e}_{i_{2}} \} \bigr\vert \\ &\quad= \bigl\vert \operatorname{Cov} \bigl(\exp \{\mathrm{i}t_{i_{1}} \tilde{e}_{i_{1}} \}, \exp \{\mathrm{i}t_{i_{2}} \tilde{e}_{i_{2}} \} \bigr) \bigr\vert \\ &\quad\leq t_{0}^{2} \Biggl\vert \sum _{q1=0}^{m}\sum_{q2=0}^{m}d_{q1} \,d_{q2}\operatorname{Cov} (e_{i_{1}+q1},e_{i_{2}+q2} ) \Biggr\vert \\ &\quad\leq -t_{0}^{2}\sum_{q1=0}^{m} \sum_{q2=0}^{m}\operatorname{Cov} (e_{i_{1}+q1},e_{i_{2}+q2} ). \end{aligned}$$

Hence, the result is true for $n=2$.

Moreover, suppose that (16) holds for $n-1$. By Lemma 3.4, we have, for *n*,

$$\begin{aligned} & \Biggl\vert E\exp \Biggl\{ \mathrm{i}\sum_{j=1}^{n}t_{i_{j}} \tilde{e}_{i_{j}} \Biggr\} -\prod_{j=1}^{n}E \exp \{\mathrm{i}t_{i_{j}}\tilde{e}_{i_{j}} \} \Biggr\vert \\ &\quad\leq \Biggl\vert E\exp \Biggl\{ \mathrm{i}\sum_{j=1}^{n}t_{i_{j}} \tilde {e}_{i_{j}} \Biggr\} -E\exp \Biggl\{ \mathrm{i}\sum _{j=1}^{n-1}t_{i_{j}}\tilde {e}_{i_{j}} \Biggr\} E\exp \{\mathrm{i}t_{i_{n}}\tilde{e}_{i_{n}} \} \Biggr\vert \\ &\qquad{}+ \Biggl\vert E\exp \Biggl\{ \mathrm{i}\sum_{i=1}^{n-1}t_{i_{j}} \tilde {e}_{i_{j}} \Biggr\} E\exp\{\mathrm{i}t_{i_{n}} \tilde{e}_{i_{n}}\}-\prod_{j=1}^{n-1}E \exp\{\mathrm{i}t_{i_{j}}\tilde{e}_{i_{j}}\}E\exp \{\mathrm {i}t_{i_{n}}\tilde{e}_{i_{n}} \} \Biggr\vert \\ &\quad \leq\Biggl\vert \operatorname{Cov} \Biggl(\exp \Biggl\{ \mathrm{i}\sum _{i=1}^{n-1}t_{i_{j}}\tilde {e}_{i_{j}} \Biggr\} ,\exp\{\mathrm{i}t_{i_{n}}\tilde{e}_{i_{n}}\} \Biggr) \Biggr\vert + \Biggl\vert E\exp \Biggl\{ \mathrm{i}\sum _{j=1}^{n-1}t_{i_{j}}\tilde {e}_{i_{j}} \Biggr\} - \prod_{j=1}^{n-1}E\exp\{ \mathrm{i}t_{i_{j}}\tilde {e}_{i_{j}}\} \Biggr\vert \\ &\quad\leq \Biggl\vert \operatorname{Cov} \Biggl(\exp \Biggl\{ \mathrm{i}\sum _{i=1}^{n-1}t_{i_{j}}\tilde {e}_{i_{j}} \Biggr\} ,\exp \{\mathrm{i}t_{i_{n}}\tilde{e}_{i_{n}} \} \Biggr) \Biggr\vert +\sum_{j=1}^{n-1}\sum _{k=j+1}^{n-1}\sum_{q1=0}^{m} \sum_{q2=0}^{m}t_{0}^{2} \bigl\vert \operatorname{Cov}(e_{i_{j}+q1},e_{i_{k}+q2}) \bigr\vert \\ &\quad \leq -t_{0}^{2}\sum_{j=1}^{n} \sum_{k=j+1}^{n}\sum _{q1=0}^{m}\sum_{q2=0}^{m}\operatorname{Cov} (e_{i_{j}+q1},e_{i_{k}+q2} ), \end{aligned}$$

which completes the proof. □

### Lemma 3.6

(Hu et al. [29])

*If Condition* (C3) *holds*, *then*

1. $|E_{0}(t,s)|\leq\frac{C_{k}}{(1+|t-s|)^{k}}$, $|E_{\tilde {m}}(t,s)|\leq\frac{2^{\tilde{m}}C}{(1+2^{\tilde{m}}|t-s|)^{k}}$ (*where* $k \in\mathbf{N} $ *and* $C=C(k)$ *is a constant depending on* *k* *only*).
2. $\sup_{0\leq s\leq1}|E_{\tilde{m}}(t,s)|=O(2^{\tilde{m}})$.
3. $\sup_{t}\int^{1}_{0}|E_{\tilde{m}}(t,s)|\,ds\leq C_{2}$.
4. $\int^{1}_{0}E_{\tilde{m}}(t,s)\,ds\rightarrow1, n\rightarrow \infty$.

### Lemma 3.7

(Rao [33])

*Suppose that* $\{X_{n}, n\geq1\}$ *are independent random variables with* $EX_{n}=0$ *and* $s_{n}^{-(2+\delta)}\sum_{j=1}^{n}E|X_{j}|^{2+\delta}\rightarrow 0$ *for some* $\delta>0$. *Then*

$$s_{n}^{-1}\sum_{j=1}^{n}X_{j} \stackrel{D}{\rightarrow} N(0,1), $$

*where* $s_{n}^{2}=\sum_{j=1}^{n}EX^{2}_{j}=\operatorname{Var} (\sum_{j=1}^{n}X_{j} )$.

### Lemma 3.8

(Yu et al. [34])

*Let* $\{e_{n}, n\geq1\}$ *be a sequence of NSD random variable satisfying* $Ee_{n}=0$, $\sup_{j\geq1}\sum_{i:|i-j|\geq u}|\operatorname{Cov}(e_{i},e_{j})|\rightarrow0$ *as* $u\rightarrow\infty$, *and* $\{a_{ni}, 1\leq i \leq n, n\geq1\}$ *be an array of real numbers with* $\max_{1\leq i\leq n}|a_{ni}|\rightarrow0$ *and* $\sum_{i=1}^{n}a^{2}_{ni}=O(1)$. *Suppose that* $\{e_{n}, n\geq1\}$ *is uniformly integral in* $L_{2}$, *then*

$$\begin{aligned} \sigma^{-1}_{n}\sum_{i=1}^{n}a_{ni}e_{i} \stackrel{D}{\rightarrow} N(0,1), \end{aligned}$$

*where* $\sigma^{2}_{n}=\operatorname{Var} (\sum_{i=1}^{n}a_{ni}e_{i} )$.

## Main results and their proofs

### Theorem 4.1

*Under Condition* (C1), *suppose that* $\{e_{n},n\geq1\}$ *is a sequence of NSD random variables with* $Ee_{n}=0$ *and*

(i) $\sup_{n\geq1}E|e_{n}|^{2+\delta}< \infty$ *for some* $\delta>0$,
(ii) $\sup_{j\geq1}\sum_{i:|i-j|\geq u}|\operatorname{Cov}(e_{i},e_{j})|\rightarrow0$ *as* $u\rightarrow\infty$. *Then*
  17 $$\begin{aligned} &(n-m)^{-\frac{1}{2}}\tau^{-1}_{\beta}\Sigma_{n}( \hat{\beta}_{n}-\beta _{0})\stackrel{D}{\longrightarrow} N(0,I_{d}) \end{aligned}$$
  *provided that*
  18 $$\begin{aligned} \tau^{2}_{\beta}=\lim_{n\rightarrow\infty}(n-m)^{-1} \Biggl\{ \sum_{i=1}^{n-m}\tilde{x}_{i} \tilde{x}^{T}_{i}\operatorname{Var} (\tilde{e}_{i} )+2\sum _{i=1}^{n-m}\sum_{j=i+1}^{n-m} \tilde{x}_{i}\tilde{x}^{T}_{j}\operatorname{Cov} ( \tilde{e}_{i},\tilde{e}_{j} ) \Biggr\} \end{aligned}$$
  *is a positive definite matrix*, *where* $I_{d}$ *is the identity matrix of order* *d*.

### Proof

By Condition (i), we have

$$\begin{aligned} \sup_{n\geq1}Ee_{n}^{2}< \infty \quad\text{and}\quad \lim_{x\rightarrow\infty }\sup_{n\geq1}Ee_{n}^{2}I \bigl\{ \vert e_{n} \vert >x\bigr\} =0, \end{aligned}$$

from which it follows that

$$\begin{aligned} C_{3}:=\sup_{n>m}{(n-m)}^{-1}\sum _{i=1}^{n-m} \sum_{q=0}^{m} \operatorname{Var} (d_{q}e_{i+q})< \infty, \end{aligned}$$

and for all $\varepsilon>0$

$$\begin{aligned} (n-m)^{-1}\sum_{i=1}^{n-m}\sum _{q=0}^{m}E(d_{q}e_{i+q})^{2}I \bigl\{ \vert d_{q}e_{i+q} \vert \geq\sqrt{n-m}\varepsilon \bigr\} \rightarrow0 \quad\text{as } n\rightarrow\infty. \end{aligned}$$

Then we can find a positive number sequence $\{\varepsilon_{n}, n\geq1\}$ with $\varepsilon_{n}\rightarrow0$ such that

$$\begin{aligned} (n-m)^{-1}\sum_{i=1}^{n-m}\sum _{q=0}^{m}E(d_{q}e_{i+q})^{2}I \bigl\{ \vert d_{q}e_{i+q} \vert \geq\sqrt{n-m} \varepsilon_{n}\bigr\} \rightarrow0 \quad\text{as } n\rightarrow\infty. \end{aligned}$$

Now, we define the integers: $m_{0}=0$, and, for each $j=0,1,2,\ldots$ , put

$$\begin{aligned} m_{2j+1}&=\min \Biggl\{ m':m'\geq m_{2j}, {(n-m)}^{-1}\sum_{i=m_{2j}+1}^{m'} \sum_{q=0}^{m}\operatorname{Var}(d_{q}e_{i+q})> \sqrt{\varepsilon _{n}} \Biggr\} , \\ m_{2j+2}&=m_{2j+1}+ \biggl\lfloor \frac{1}{\varepsilon_{n}} \biggr\rfloor +m. \end{aligned}$$

Denote

$$\begin{aligned} I_{j}&=\{k:m_{2j}< k\leq m_{2j+1}, j=0, \ldots, l \} \quad\text{and} \\ J_{j}&=\{k:m_{2j+1}< k\leq m_{2(j+1)}, j=0, \ldots, l \}, \end{aligned}$$

where $l=l(n)$ is the number of blocks of indices $I_{j}$. Then

19 $$\begin{aligned} l\sqrt{\varepsilon_{n}}\leq{(n-m)}^{-1}\sum _{j=1}^{l}\sum_{i\in I_{j}} \sum _{q=0}^{m} \operatorname{Var} (d_{q}e_{i+q}) \leq{(n-m)}^{-1}\sum_{i=1}^{n-m} \sum _{q=0}^{m} E(d_{q}e_{i+q})^{2} \leq C_{3}, \end{aligned}$$

and hence we have $l\leq C_{3}/\sqrt{\varepsilon_{n}}$. If the number of the remainder term is not zero when the construction ends, then we put all the remainder terms into a block denoted by $J_{l}$. By (7), we have

20 $$\begin{aligned} \Sigma_{n}(\hat{\beta}_{n}-\beta_{0})=\sum _{i=1}^{n-m}\tilde{x}_{i} \tilde{e}_{i}. \end{aligned}$$

Then to prove (17), it is enough to prove that

21 $$\begin{aligned} (n-m)^{-1/2}\tau^{-1}_{\beta}\sum _{i=1}^{n-m}\tilde{x}_{i}\tilde {e}_{i}\stackrel{D}{\rightarrow} N(0,I_{d}). \end{aligned}$$

Let *u* be an arbitrary *d*-dimensional column vector with $\|u\|=1$, and set $a_{i}=u^{T}\tau^{-1}_{\beta}\tilde{x}_{i}$. Then, by the Cramér–Wold device, to prove (21) it suffices to prove that

22 $$\begin{aligned} \frac{1}{\sqrt{n-m}}\sum_{i=1}^{n-m}a_{i} \tilde{e}_{i}\stackrel{D}{\rightarrow} N(0,1). \end{aligned}$$

Write

$$\begin{aligned} \frac{1}{\sqrt{n-m}}\sum_{i=1}^{n-m}a_{i} \tilde{e}_{i} &= \frac{1}{\sqrt{n-m}}\sum_{j=1}^{l} \sum_{i\in I_{j}}a_{i}\tilde{e}_{i}+ \frac {1}{\sqrt{n-m}}\sum_{j=1}^{l}\sum _{i\in J_{j}}a_{i}\tilde{e}_{i} \\ &:=I+J. \end{aligned}$$

Moreover, note that $\max_{0\leq q\leq m}|d_{q}|\leq1$ and $\max_{1\leq i\leq n}|a_{i}|<\infty$ by Condition (C1), then applying Lemma 3.3 with $p=2$ we have

23 $$\begin{aligned} & E \Biggl(\frac{1}{\sqrt{n-m}}\sum_{j=1}^{l} \sum_{i \in J_{j}}a_{i}\tilde {e}_{i} \Biggr)^{2} \\ &\quad= \frac{1}{n-m}E \Biggl(\sum_{j=1}^{l} \sum_{i\in J_{j}}\sum_{q=0}^{m}a_{i}d_{q}e_{i+q} \Biggr)^{2} \\ &\quad\leq\frac{4m}{n-m} \sum_{j=1}^{l}\sum _{i\in J_{j}}\sum_{q=0}^{m}E \vert a_{i}d_{q}e_{i+q} \vert ^{2} \\ &\quad\leq\frac{4m}{n-m} \Bigl(\max_{m_{1}\leq i\leq m_{2l+2}}a_{i}^{2} \Bigr) \sum_{j=1}^{l}\sum _{i\in J_{j}}\sum_{q=0}^{m}E \vert d_{q}e_{i+q} \vert ^{2} \\ &\quad\leq\frac{4m}{n-m} \Bigl(\max_{m_{1}\leq i\leq m_{2l+2}}a_{i}^{2} \Bigr)\sum_{j=1}^{l}\sum _{i\in J_{j}}\sum_{q=0}^{m}E \vert d_{q}e_{i+q} \vert ^{2}I \bigl\{ \vert d_{q}e_{i+q} \vert \geq\sqrt{n-m}\varepsilon_{n} \bigr\} \\ &\qquad{}+\frac{4m}{n-m} \Bigl(\max_{m_{1}\leq i\leq m_{2l+2}}a_{i}^{2} \Bigr)\sum_{j=1}^{l}\sum _{i\in J_{j}}\sum_{q=0}^{m}E \vert d_{q}e_{i+q} \vert ^{2}I \bigl\{ \vert d_{q}e_{i+q} \vert < \sqrt{n-m}\varepsilon_{n} \bigr\} \\ &\quad\leq\frac{4m}{n-m} \Bigl(\max_{m_{1}\leq i\leq m_{2l+2}}a_{i}^{2} \Bigr) \sum_{j=1}^{l}\sum _{i\in J_{j}}\sum_{q=0}^{m}E \vert d_{q}e_{i+q} \vert ^{2}I \bigl\{ \vert d_{q}e_{i+q} \vert \geq\sqrt{n-m}\varepsilon_{n} \bigr\} \\ &\qquad{}+\frac{4m}{n-m} \Bigl(\max_{m_{1}\leq i\leq m_{2l+2}}a_{i}^{2} \Bigr)l\bigl(\bigl\lfloor \varepsilon^{-1}_{n}\bigr\rfloor +m \bigr) (n-m)\varepsilon^{2}_{n} \\ &\quad\leq\frac{4m}{n-m} \Bigl(\max_{m_{1}\leq i\leq m_{2l+2}}a_{i}^{2} \Bigr)\sum_{i=1}^{n-m}\sum _{q=0}^{m}E(d_{q}e_{i+q})^{2}I \bigl\{ \vert d_{q}e_{i+q} \vert \geq \sqrt{n-m} \varepsilon_{n}\bigr\} \\ &\qquad{}+4m \Bigl(\max_{m_{1}\leq i\leq m_{2l+2}}a_{i}^{2} \Bigr)C_{3}{\varepsilon _{n}}^{-1/2}\bigl(\bigl\lfloor \varepsilon^{-1}_{n}\bigr\rfloor +m\bigr) \varepsilon^{2}_{n} \\ &\quad\rightarrow 0\quad \text{as } n\rightarrow\infty, \end{aligned}$$

which follows from

$$\begin{aligned} J \stackrel{P}{\rightarrow} 0 \quad\text{as } n\rightarrow\infty \end{aligned}$$

by the Markov inequality. Therefore, to prove (22), it suffices to show that

24 $$\begin{aligned} \frac{1}{\sqrt{n-m}}\sum_{j=1}^{l}\sum _{i\in I_{j}}a_{i}\tilde{e}_{i} \stackrel{D}{\rightarrow} N(0,1). \end{aligned}$$

On the one hand, by the definition of $\tau^{2}_{\beta}$, it is easy to show that

$$\begin{aligned} \lim_{n\rightarrow\infty}\operatorname{Var} \Biggl(\frac{1}{\sqrt{n-m}}\sum _{i=1}^{n-m}a_{i}\tilde{e}_{i} \Biggr)=1. \end{aligned}$$

Therefore by the above formula and (23),

25 $$\begin{aligned} \lim_{n\rightarrow\infty}\operatorname{Var} \Biggl(\frac{1}{\sqrt{n-m}}\sum _{j=1}^{l}\sum_{i\in I_{j}}a_{i} \tilde{e}_{i} \Biggr)=1. \end{aligned}$$

On the other hand, by Lemma 3.5 and (ii), we have

26 $$\begin{aligned} & \Biggl\vert E\exp \Biggl(i\sum_{j=1}^{l} \sum_{i\in I_{j}}t_{i}\tilde{e}_{i} \Biggr)-\prod^{l}_{j=1}E \biggl(\sum _{i\in I_{j}}\exp(it_{i}\tilde{e}_{i}) \biggr) \Biggr\vert \\ &\quad\leq -t_{0}^{2}\sum^{l}_{p=1} \sum^{l}_{s=p+1}\sum _{i\in I_{p}}\sum_{j\in I_{s}}\sum _{q1=0}^{m} \sum_{q2=0}^{m} \operatorname{Cov} (e_{i+q1}, e_{j+{q2}} ) \\ &\quad= -t_{0}^{2}\sum_{q1=0}^{m} \sum_{q2=0}^{m} \sum _{i+q1-j-{q2}\geq\lfloor\frac {1}{\varepsilon_{n}}\rfloor+m}\operatorname{Cov}(e_{i+q1}, e_{j+{q2}}) \\ &\quad\rightarrow 0\quad \text{as } n\rightarrow\infty, \end{aligned}$$

which implies that the problem now is reduced to study the asymptotic behavior of independent and non-identically distribution random variables $\{\sum_{i\in I_{j}}a_{i}\tilde{e}_{i} \}$.

To complete the proof of (24), it is enough to show that random variables $\{\sum_{i\in I_{j}}a_{i}\tilde{e}_{i} \}$ satisfies the condition of Lemma 3.7. Set

$$C_{4}=\max_{1\leq i \leq m_{2l+2}} \vert a_{i} \vert ^{2+\delta} \quad\text{and} \quad\tau_{n}^{2}= \operatorname{Var} \Biggl( \frac{1}{\sqrt{n-m}}\sum_{i=1}^{n-m}a_{i} \tilde {e}_{i} \Biggr). $$

By the definition of $I_{j}$,

27 $$\begin{aligned} & (n-m)^{-1}\sum_{i\in I_{j}}\sum ^{m}_{q=0}E(d_{q}e_{i+q})^{2} \\ &\quad= (n-m)^{-1}\sum^{m_{2j+1}}_{m_{2j}}\sum ^{m}_{q=0}E(d_{q}e_{i+q})^{2} \\ &\quad = (n-m)^{-1}\sum^{m_{2j+1}-1}_{m_{2j}}\sum ^{m}_{q=0}E(d_{q}e_{i+q})^{2}+(n-m)^{-1} \sum^{m}_{d=0}E(d_{q}e_{m_{2j+1}+q})^{2} \\ &\quad\leq \sqrt{\varepsilon_{n}}+(n-m)^{-1}\sum ^{m}_{q=0}E(d_{q}e_{m_{2j+1}+q})^{2} \\ &\quad\leq \sqrt{\varepsilon_{n}}+(n-m)^{-1}\sup _{n\geq1}Ee_{n}^{2}. \end{aligned}$$

By Lemma 3.3 with $p=2+\delta$ and (27), and recalling that $l\leq C_{3}/\sqrt{\varepsilon_{n}}$,

28 $$\begin{aligned} & \tau_{n}^{-(2+\delta)}\sum_{j=1}^{l}E \biggl\vert (n-m)^{-1/2}\sum_{i\in I_{j}}a_{i} \tilde{e}_{i} \biggr\vert ^{2+\delta} \\ &\quad= \tau_{n}^{-(2+\delta)}(n-m)^{-(2+\delta)/2}\sum _{j=1}^{l}E \Biggl\vert \sum _{i\in I_{j}}\sum^{m}_{q=0}a_{i}d_{q}e_{i+q} \Biggr\vert ^{2+\delta} \\ &\quad \leq\tau_{n}^{-(2+\delta)}(n-m)^{-(2+\delta)/2}C_{4}2^{\delta+3}m^{\delta +1} \biggl(\frac{15(2+\delta)}{\ln(2+\delta)} \biggr)^{2+\delta}\sum_{j=1}^{l} \sum_{i\in I_{j}}\sum_{q=0}^{m}E \vert d_{q}e_{i+q} \vert ^{2+\delta } \\ &\qquad{}+\tau_{n}^{-(2+\delta)}C_{4}2^{\delta+3}m^{\delta+1} \biggl(\frac {15(2+\delta)}{\ln(2+\delta)} \biggr)^{2+\delta}\sum _{j=1}^{l} \Biggl\{ (n-m)^{-1}\sum _{i\in I_{j}}\sum^{m}_{q=0}E(d_{q}e_{i+q})^{2} \Biggr\} ^{(2+\delta )/2} \\ &\quad \leq \tau_{n}^{-(2+\delta)}(n-m)^{-\delta/2}C_{4}2^{\delta+3}m^{\delta +2} \biggl(\frac{15(2+\delta)}{\ln(2+\delta)} \biggr)^{2+\delta}\sup_{n\geq1}E \vert e_{n} \vert ^{2+\delta} \\ &\qquad{}+\tau_{n}^{-(2+\delta)}C_{4}2^{\delta+3}m^{\delta+1} \biggl(\frac {15(2+\delta)}{\ln(2+\delta)} \biggr)^{2+\delta}\cdot C_{3}\varepsilon _{n}^{-1/2} \Bigl\{ \sqrt{\varepsilon_{n}}+(n-m)^{-1} \sup_{n\geq 1}Ee_{n}^{2} \Bigr\} ^{(2+\delta)/2} \\ &\quad\rightarrow 0, \end{aligned}$$

since $\tau_{n}\rightarrow1$ and (i).

Hence, by Lemma 3.7, (24) holds and the proof is completed. □

### Corollary 4.1

*Under Condition* (C1), *let* $\{e_{n},n\geq1\}$ *be a sequence of independent random variables with* $Ee_{n}=0$, *and suppose that* (i) *of Theorem *4.1 *holds and* $Ee^{2}_{n}=\sigma^{2}$ *for all* $n\geq1$. *Then*

$$\begin{aligned} &(n-m)^{-\frac{1}{2}}\tau^{-1}_{\beta}\Sigma_{n}( \hat{\beta}_{n}-\beta _{0})\stackrel{D}{\longrightarrow} N(0,I_{d}), \end{aligned}$$

*provided that*

$$\tau^{2}_{\beta}=\lim_{n\rightarrow\infty}(n-m)^{-1} \Biggl\{ \sum_{i=1}^{n-m}\tilde{x}_{i} \tilde{x}^{T}_{i} \sigma^{2}+2\sum _{k=1}^{m}\sum_{i=1}^{n-m-k} \tilde{x}_{i}\tilde {x}^{T}_{i+k} (d_{0}d_{k}+d_{1}d_{k}+ \cdots+d_{m-k}d_{m} )\sigma^{2} \Biggr\} $$

*is a positive definite matrix*.

### Proof

Since $\{e_{n},n\geq1\}$ is a sequence of independent random variables, we have $\operatorname{Cov}(e_{i}, e_{j})=0$ if $i\neq j$ and hence $\operatorname{Cov}(\tilde{e}_{i},\tilde {e}_{j})=0$ if $|i-j|>m$. It follows that

29 $$\begin{aligned} \tau^{2}_{\beta}={}& \lim_{n\rightarrow\infty}(n-m)^{-1} \Biggl\{ \sum_{i=1}^{n-m}\tilde{x}_{i} \tilde{x}^{T}_{i}\operatorname{Var} (\tilde{e}_{i} )+\sum _{i=1}^{n-m}\sum_{j=1,j\neq i}^{n-m} \tilde{x}_{i}\tilde{x}^{T}_{j}\operatorname{Cov} ( \tilde{e}_{i},\tilde{e}_{j} ) \Biggr\} \\ ={}& \lim_{n\rightarrow\infty}(n-m)^{-1} \Biggl\{ \sum _{i=1}^{n-m}\tilde {x}_{i} \tilde{x}^{T}_{i}\sigma^{2}+2\sum _{k=1}^{m}\sum_{i=1}^{n-m-k} \tilde {x}_{i}\tilde{x}^{T}_{i+k} (d_{0}d_{k}+d_{1}d_{k+1}+ \cdots \\ &{}+d_{m-k}d_{m} )\sigma^{2} \Biggr\} \end{aligned}$$

from the conditions of Corollary 4.1, we see that $\tau^{2}_{\beta}$ is a positive definite matrix. Thus the result follows from (29). □

### Theorem 4.2

*Assume the conditions of Theorem *4.1, *and further assume that Conditions* (C2)–(C5) *hold*. *Then*

30 $$\begin{aligned} \sup_{0\leq t \leq1} \bigl\vert \hat{f}_{n}(t)-f(t) \bigr\vert =O_{P}\bigl(n^{-\gamma }\bigr)+O_{P}( \tau_{\tilde{m}})+O_{P}\bigl(n^{-1/3}M_{n}\bigr)\quad \textit{as }n\rightarrow\infty, \end{aligned}$$

*where* $M_{n}\rightarrow\infty$ *in arbitrary slowly rate*, *and* $\tau _{\tilde{m}}=2^{-\tilde{m}(\alpha-1/2)}$ *if* $1/2< \alpha<3/2$, $\tau _{\tilde{m}}= \sqrt{\tilde{m}}2^{-\tilde{m}}$ *if* $\alpha=3/2$, *and* $\tau_{\tilde {m}}=2^{-\tilde{m}}$ *if* $\alpha>3/2$.

### Proof

We can prove Theorem 4.2 by a similar argument to Theorem 3.2 of Hu et al. [12], so we omit the detail. □

### Theorem 4.3

*Under the Conditions of Theorem *4.2, *we have*

31 $$\begin{aligned} \frac{\hat{f}_{n}(t)-f(t)}{\tau_{t}}\stackrel{D}{\rightarrow} N(0,1), \end{aligned}$$

*where* $\tau^{2}_{t}=\operatorname{Var} (\sum_{i=1}^{n}e_{i}\int_{A_{i}}{E_{\tilde{m}}(t,s)\, ds} )$.

### Proof

Note

32 $$\begin{aligned} \hat{f}_{n}(t)-f(t)={}& \sum^{n}_{i=1} \bigl(y_{i}-x^{T}_{i}\hat{\beta}_{n} \bigr) \int_{A_{i}}E_{\tilde {m}}(t,s)\,ds-f(t) \\ ={}& \sum^{n}_{i=1} \bigl(x^{T}_{i} \beta+f(t_{i})+e_{i}-x^{T}_{i}\hat{ \beta}_{n} \bigr) \int _{A_{i}}E_{\tilde{m}}(t,s)\,ds-f(t) \\ ={}& \sum^{n}_{i=1}x^{T}_{i} (\beta-\hat{\beta}_{n} ) \int_{A_{i}}E_{\tilde {m}}(t,s)\,ds \\ &{}+ \Biggl\{ \sum^{n}_{i=1}f(t_{i}) \int_{A_{i}}E_{\tilde{m}}(t,s)\,ds-f(t) \Biggr\} +\sum ^{n}_{i=1}e_{i} \int_{A_{i}}E_{\tilde{m}}(t,s)\,ds \\ :={}&I_{1}+I_{2}+I_{3}, \end{aligned}$$

from the proof of Theorem 3.2 in Hu et al. [12], we get $I_{1}=O_{P}(n^{-1/2})$, $I_{2}=O_{P}(n^{-\gamma})+O_{P}(\tau_{\tilde{m}})$ and $I_{3}=O_{P}(n^{-1/3}M_{n})$, and it implies that

$$I_{1}=o_{P}(I_{3}) $$

and

$$I_{2}=o_{P}(I_{3}). $$

Then we should prove

33 $$\begin{aligned} \frac{I_{3}}{\tau_{t}}=\frac{\sum^{n}_{i=1}e_{i}\int_{A_{i}}E_{\tilde{m}}(t,s)\, ds}{\sqrt{\operatorname{Var} (\sum^{n}_{i=1}e_{i}\int_{A_{i}}E_{\tilde{m}}(t,s)\,ds )}}\stackrel{D}{\rightarrow} N(0,1). \end{aligned}$$

Let $a_{ni}=\tau^{-1}_{t}\int_{A_{i}}E_{\tilde{m}}(t,s)\,ds$, then, by Lemma 3.6 and (C5), $\max_{1\leq i\leq n}|a_{ni}|\rightarrow0$, and $\sum_{i=1}^{n}a^{2}_{ni}=O(1)$, and condition (i) implies that $\{e_{n}, n\geq1\}$ is a uniformly integral family on $L_{2}$, then, by Lemma 3.8 and (ii), we have

34 $$\begin{aligned} \tau^{-1}_{t} \bigl(\hat{f}_{n}(t)-f(t) \bigr) \stackrel{D}{\rightarrow} N(0,1). \end{aligned}$$

The proof is completed. □

## A simulation example

In this section, we perform a simulation example to verify the accuracy of Theorem 4.1 and Theorem 4.3. Consider the partially linear model

$$y_{i}=x_{i}\beta+f(t_{i})+e_{i}, \quad i=1, 2, \ldots, n, $$

where $x_{i}=\cos(2\pi t_{i}), f(t_{i})=\sin(2\pi t_{i}),\beta_{0}=5, t_{i}=i/n$, $e_{i}$ is NSD sequence and raised as follows.

Let $\{e_{1}, e_{2}, \ldots, e_{n}\}$ be a sequence of independent and identically distributed random variables with common probability mass function $P(e_{1}=0)=2P(e_{1}=1)=P(e_{1}=2)=0.4$. Then $\{e_{1},e_{2},\ldots,e_{n}\}$ given $S_{n}=n$ is NSD by Theorem 3.1 in Hu [25], where $S_{n}=\sum_{i=1}^{n}e_{i}$.

**Figure 1:**
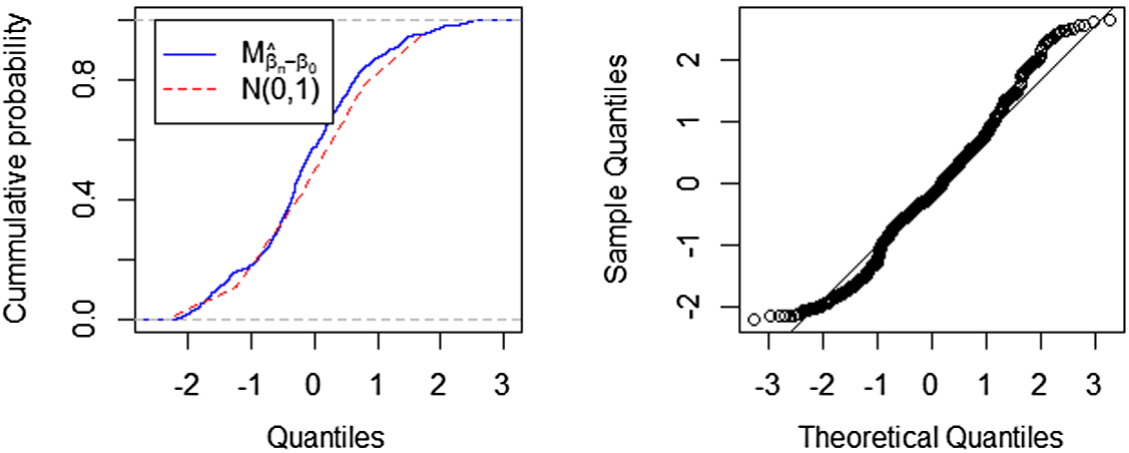
A comparison fitted distribution functions of $M_{\hat{\beta }_{n}-\beta_{0}}$ and $N(0,1)$, and QQ-plot of $M_{\hat{\beta}_{n}-\beta_{0}}$, where $n=64$

**Figure 2:**
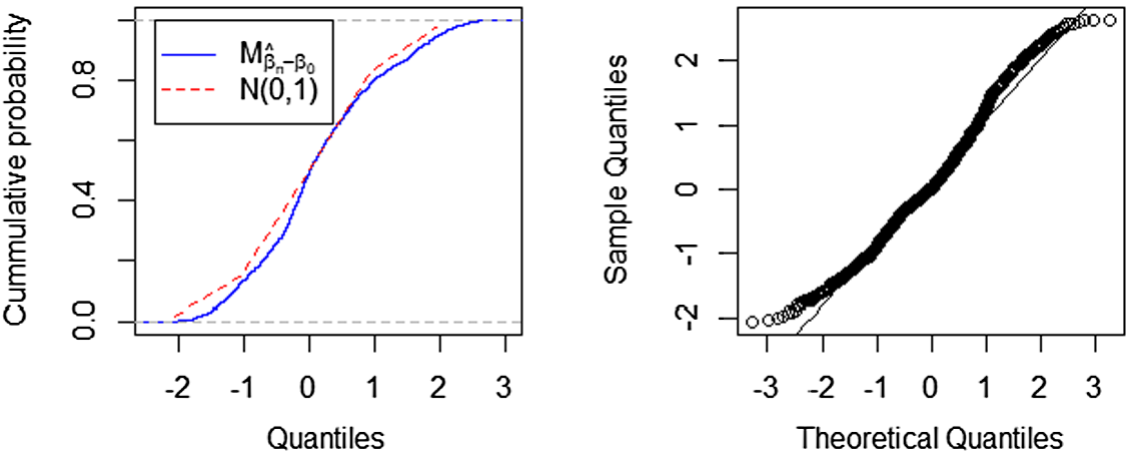
A comparison fitted distribution functions of $M_{\hat{\beta }_{n}-\beta_{0}}$ and $N(0,1)$, and QQ-plot of $M_{\hat{\beta}_{n}-\beta_{0}}$, where $n=128$

Set $m=3$ and the difference sequence $d_{0}=\sqrt {3/4},d_{1}=d_{2}=d_{3}=-\sqrt{1/12}$ (Wang et al. [9]). We first evaluate the $M_{\hat{\beta}_{n}-\beta_{0}}=(n-m)^{-\frac{1}{2}}\tau ^{-1}_{\beta}\Sigma_{n} (\hat{\beta}_{n}-\beta_{0} )$ approximation. Figures 1 and 2 show the results for two sample size specifications ($n=64, n=128$). Panel 1 in Fig. 1 compares the empirical distribution functions of $M_{\hat{\beta}_{n}-\beta_{0}}$ and $N(0,1)$. Panel 2 in Fig. 1 gives the QQ-plot of $M_{\hat{\beta }_{n}-\beta_{0}}$. Figure 1 shows that the distribution of $M_{\hat{\beta}_{n}-\beta_{0}}$ can approximate $N(0,1)$ well even if the sample size are not large ($n=64$). Comparison of Fig. 2 with Fig. 1 indicates that the distribution approximation for the larger sample size is much more accurate than that for the small one.

**Figure 3:**
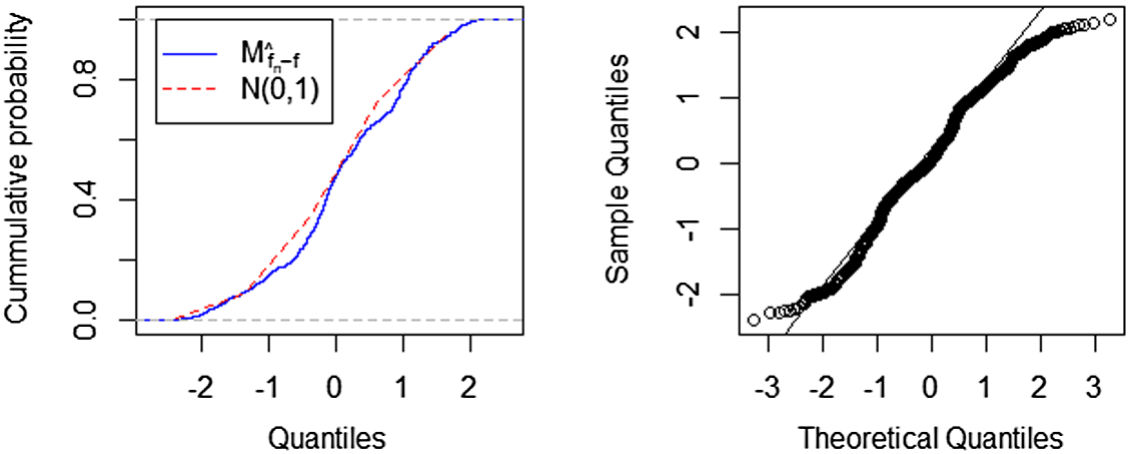
A comparison fitted distribution functions of $M_{\hat{f}_{n}-f}$ and $N(0,1)$, and QQ-plot of $M_{\hat{f}_{n}-f}$, where $n=64$

**Figure 4:**
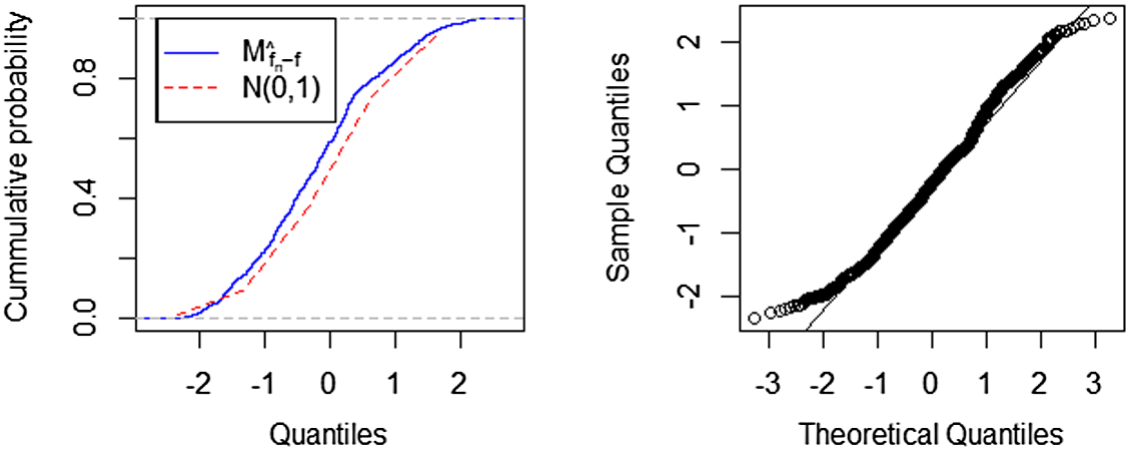
A comparison fitted distribution functions of $M_{\hat{f}_{n}-f}$ and $N(0,1)$, and QQ-plot of $M_{\hat{f}_{n}-f}$, where $n=128$

Choose the Daubechies scaling function $_{2}\phi(t)$ as in Hu et al. [29]. Figures 3 and 4 show that the distribution of $M_{\hat{f}_{n}-f}=\tau^{-1}_{t} (\hat{f}_{n}(t)-f(t) )$ is closer and closer to $N(0,1)$ with the increasing sample size.

## Conclusions

In this paper, we use a difference-based and ordinary least square (DOLS) method to obtain the estimator of the unknown parametric component *β* of the partial linear model with dependent errors. In addition, we investigate the asymptotic normality for the DOLS estimator of *β* and wavelet estimator of $f(\cdot)$. Thus, we extend some results of Hu et al. [12] to the partially linear model with NSD errors. Furthermore, NSD random variables contain negatively associated random variables. Therefore, it is an interesting subject to investigate the limit properties of the difference-based estimator for a partially linear model with NSD errors in future studies.
